# An acute need: precision medicine for acute care surgery

**DOI:** 10.1093/bjsopen/zrad003

**Published:** 2023-03-08

**Authors:** Rajarshi Mukherjee

**Affiliations:** Liverpool EmerGenT Academy, Emergency General & Major Trauma Surgery, Aintree University Hospital, Liverpool University Hospitals NHS Foundation Trust, Fazakerley, Liverpool, UK; Institute of Systems, Molecular and Integrative Biology, University of Liverpool, Liverpool, UK

The premise of precision medicine is not new. Over 100 years ago, Sir William Osler, the great Canadian physician, said ‘*The good physician treats the disease; the great physician treats the patient who has the disease*’^1^. These wise and visionary words reflect the essential essence of what precision medicine is all about: patient-tailored therapeutic interventions utilizing genetic and molecular profiling to maximize patient benefit. Indeed, recent surveys amongst members of the Royal College of Physicians have highlighted how improved patient stratification and characterization to facilitate precision medicine is consistently reported as a key focus for the future of acute and emergency care^2^. Disappointingly, acute care surgeons have been poor in implementing these advances for their patients, with a recent global survey assessing publications and trials in the field of precision medicine identifying a concerning widening gap in research and translation in acute care surgery when compared with surgical oncology and acute medicine^3^.

To address the widening gap in translation, multiple groups of acute care surgeons have now highlighted the burgeoning need that exists for precision medicine in acute care surgery, with significant progress in conditions such as sepsis, trauma, and pancreatitis^4–6^. This is even more relevant considering how critical illness is being redefined, moving away from syndromic definitions to a focus on the overlap and uniqueness of key underlying biological changes^7^. Undeniably, multiple areas of potential benefit for precision medicine exist in acute care surgery: enhanced point-of-care diagnostics to improve outcomes in ambulatory surgery; improved and earlier diagnosis in difficult-to-diagnose conditions such as bowel ischaemia; better patient prognostication for an ageing population undergoing emergency laparotomy; and more efficacious personalized critical care management for a wide range of acute inflammatory conditions including abdominal sepsis, polytrauma, and pancreatitis.

Future progress requires us to address the over-simplistic criticisms that are used to justify the sluggish implementation of precision medicine in acute care surgery^8^. Some cite the very definition of the word acute, ‘severe but of a short duration’, to stress that precision medicine takes too long or is too expensive to have a justifiable impact on the emergency field. These arguments fail to understand the pace of change and progress, with DNA sequencing being the perfect example of this. It originally cost €930 000 000 to sequence a single human genome in 1990, whilst Illumina, a major international DNA sequencing company, recently reported a landmark drop to below €100, signposting a ‘game-changing’ piece of progress facilitating a new era of personalized medicine^9^. Similarly, new rapid PCR-based diagnostic technologies, taking only 45 min to run and providing clinically actionable data, have been validated for a range of respiratory conditions including COVID-19 and now hold immense potential for acute abdominal conditions^10^.

Ultimately, to provide our patients in acute care surgery with the improvements in care they rightly deserve, a paradigm shift in ideology is required. Surgical ideologies of the past, built solely on the understanding of general anatomy and relying on the inheritance of surgical dogma, must be replaced with broader new mindsets. These mindsets must be based on the ongoing understanding of individual biological variation and the propagation of surgical precision instead. It is high time for acute care surgeons to change the age-old surgical aphorism ‘when in doubt, cut it out’ to ‘when in doubt, learn more about’.
